# Lassa Virus Treatment Options

**DOI:** 10.3390/microorganisms9040772

**Published:** 2021-04-07

**Authors:** Frederick Hansen, Michael A. Jarvis, Heinz Feldmann, Kyle Rosenke

**Affiliations:** 1 Laboratory of Virology, Division of Intramural Research, National Institute of Allergy and Infectious Diseases, National Institutes of Health, Hamilton, MT 59840, USA; frederick.hansen@nih.gov (F.H.); feldmannh@niaid.nih.gov (H.F.); 2 The Vaccine Group Ltd., University of Plymouth, Plymouth PL4 8AA, UK; michael.jarvis@plymouth.ac.uk

**Keywords:** Lassa, LASV, antiviral, therapeutic, antibody, animal model

## Abstract

Lassa fever causes an approximate 5000 to 10,000 deaths annually in West Africa and cases have been imported into Europe and the Americas, challenging public health. Although Lassa virus was first described over 5 decades ago in 1969, no treatments or vaccines have been approved to treat or prevent infection. In this review, we discuss current therapeutics in the development pipeline for the treatment of Lassa fever, focusing on those that have been evaluated in humans or animal models. Several treatments, including the antiviral favipiravir and a human monoclonal antibody cocktail, have shown efficacy in preclinical rodent and non-human primate animal models and have potential for use in clinical settings. Movement of the promising preclinical treatment options for Lassa fever into clinical trials is critical to continue addressing this neglected tropical disease.

## 1. Introduction

Lassa virus (LASV), a member of the *Mammarenavirus* genus in the *Arenaviridae* family, is the causative agent of Lassa fever (LF). LASV was originally isolated and described in 1969 after a missionary nurse in Lassa, Nigeria became infected and died from the disease [1]. LASV, similar to other arenaviruses, is a negative-strand RNA virus whose enveloped virions are pleiomorphic in nature and range from 40 to 300 nm in diameter [2,3]. Arenavirus genomes consist of two ambisense single-stranded RNA segments referred to as the small (S) and large (L) segments. The 7.2 kb L segment encodes both the viral RNA-dependent RNA polymerase (RdRp) as well as the zinc-binding protein. The 3.4 kb S segment encodes the glycoprotein precursor complex (GPC) along with the nucleoprotein [4]. The GPC is co- and post-translationally cleaved into the signal peptide, GP1, and GP2.

The natural reservoir of LASV is the peridomestic multimammate rodent, *Mastomys natalensis* (Mastomys). Mastomys are distributed throughout sub-Saharan Africa with multiple identified phylogroups throughout their extensive range [5]. Recent studies have also implicated the rodent species *Hylomyscus pamfi* and *Mastomys erthrocyclus* as additional reservoirs of LASV, but their impact on overall disease burden is currently undetermined [6]. LASV spillover from Mastomys into humans is thought to occur via many routes, including direct contact with rodent excreta, inhalation of aerosols containing rodent excreta, through rodent bites, and through rodent handling and consumption [7,8]. Incidence rates of LASV have been correlated to seasonal changes, specifically rainfall, which is believed to correspond to alterations in the interaction between Mastomys and humans [9,10]. Direct human to human transmission, including cases of nosocomial transmission, have also been observed through exposure to the virus stemming from contact with the blood or other bodily fluids from infected individuals [7,10,11,12,13].

An approximate 300,000 to 500,000 LASV infections with an associated 5000 to 10,000 deaths, occur annually across sub-Saharan west Africa, with the vast majority of viral burden occurring in Nigeria, Sierra Leone, Liberia, and Guinea [9,14,15]. Consistent with these numbers, it is estimated that 80% of infections result in sub-clinical infection or mild illness, while 20% of infections result in more severe disease that require hospitalization [7]. The case fatality rate from severe/hospitalized cases reaches 15%, with the overall case fatality rate of LF being about 1% [7,12]. The incubation period for LF ranges from 6–21 days. Symptoms of LASV infection can be non-specific and LF is often only considered as a potential cause of illness after exclusion of other diseases such as typhoid fever and malaria. Early clinical symptoms include weakness, malaise, fever, sore throat, body pains, nausea, vomiting, diarrhea, and cough [7,9]. Late stage clinical manifestations include mucosal and internal bleeding, seizures, coma, disorientation, and deafness. Patients typically succumb to disease within 14 days of initial symptom onset [9].

Currently, off label use of ribavirin, fluid replacement, and dialysis are used for treatment of severe LF [16,17]. Since its initial identification in 1969 about 30 cases of exported LASV have been reported in 9 non-endemic countries. LASV therefore represents a serious exposure risk to healthcare workers and a significant public health concern worldwide [18]. Because of its epidemic potential and the current lack of approved vaccines or treatments, LASV was added to the WHO List of Blueprint Priority Diseases/Pathogens in 2018. Together, the substantial disease burden in endemic countries and continued threat from LASV exportation to non-endemic regions emphasizes the need for a maintained effort to develop countermeasures for LASV and to prepare for potential outbreaks. This review will discuss antivirals currently in use or under investigation for treatment of LASV infection, focusing on those therapeutics that have already been tested in preclinical animal models or humans.

Abbreviations are summarized in Supplementary Table S1.

## 2. Preclinical Models

Several animal models have been developed to investigate LASV disease and pathogenesis and have demonstrated differing utility for testing of therapeutic countermeasures against the virus. The pros and cons of these models, which include guinea pigs, mice, and non-human primates (NHPs), have recently been reviewed [19,20]. Inbred Strain 13 and outbred Hartley guinea pigs are considered the small animal models of choice when studying LASV [19]. Both guinea pig strains are susceptible to wildtype LASV infection via multiple infection routes with Strain 13 exhibiting a case fatality rate close to 100%, compared to Hartley guinea pigs, which show a case fatality rate closer to 30% [21,22,23]. A guinea pig adapted LASV (strain Josiah) has been developed and infection results in 100% lethality in Hartley guinea pigs [24]. Several immunocompromised mouse strains have also been developed that show susceptibility to LASV infection. These mouse strains include interferon alpha receptor knock-out (IFNAR‒/‒), human/mouse-chimeric HLA-A2.1 (humanized HHD), chimeric IFNA-/-B6, CBA, and STAT deficient (STAT1-/- mice), which show varying manifestations of LASV disease ranging from semi to fully lethal [19,20,25]. Although rhesus macaques, common marmosets, and squirrel monkeys have been described as LASV models, the most frequently used NHP model is the cynomolgus macaque with disease manifestations closely mimicking that of severe LF in humans. Disease severity in this NHP model is dependent on LASV dose and strain [19,26,27].

## 3. Antiviral Approaches

### 3.1. Ribavirin (1-[(2R,3R,4S,5R)-3,4-dihydroxy-5-(hydroxymethyl)oxolan-2-yl]-1,2,4-triazole-3-carboxamide)

Ribavirin, a guanosine analog with broad spectrum antiviral activity, was first synthesized in 1972 and has shown efficacy against both DNA and RNA viruses (Supplementary Table S2) [28]. Since its original synthesis, ribavirin has been tested for efficacy against multiple viruses including respiratory syncytial virus, HIV, influenza, measles, bunyaviruses, and arenaviruses, including Lassa virus, with mixed results [29,30,31,32,33,34,35,36]. Ribavirin is most commonly used in combination for the treatment of chronic hepatitis C virus infection, but the drug has also been used as an off-label treatment for LF, which is based primarily on results from a single clinical trial from 1986 that has recently been disputed [36,37,38,39]. Although generally well tolerated, reversible hemolytic anemia has been identified as a common side effect of therapy, which may require dosage adjustment or treatment discontinuation in severe cases [40,41].

#### 3.1.1. Mechanism of Action

Ribavirin is thought to function through multiple distinct mechanisms. The ribavirin metabolite, ribavirin monophosphate (RMP), has been proposed as one of the primary active forms of the drug [42]. RMP has been shown to inhibit the activity of inosine monophosphate dehydrogenase (IMPDH), a catalyst in the synthesis of guanosine triphosphate (GTP), resulting in the disruption of critical viral replication steps [42,43,44,45]. Additionally, ribavirin triphosphate (RTP) as an additional ribavirin metabolite, has been demonstrated to act as a nucleoside analog and exhibit mutagenic activity through its incorporation into the viral genome by the viral RNA polymerase. This accumulation of mutations within the virus genome is believed to result in inhibition of virus replication through a phenomenon called ‘genomic catastrophe’ [46,47]. RMP and RTP have been postulated to work synergistically, with RMP decreasing the availability of GTP through IMPDH inhibition and thereby lowering competition for inclusion of the mutagenic RTP by the viral RNA polymerase [46]. Rather than disrupting viral replication, the antiviral effect of ribavirin may also be attributable to the drug reducing cell death of LASV infected cells, with the drug potentially exerting these effects through the inhibition of macrophage activation, cytokine production, and lymphocyte proliferation [48].

#### 3.1.2. Preclinical Studies

Since its initial formulation, ribavirin has consistently shown its efficacy against LASV in vitro within a variety of cell types and was first tested in vivo in a rhesus macaque model in 1979 [49,50]. Rhesus macaques were treated with ribavirin with a loading dose of 50 mg/kg initiated on 0 or 5 days post-infection (dpi) followed by three times daily doses of 10 mg/kg delivered intramuscularly (Table 1) [50]. Animals were challenged subcutaneously with 10,000 pfu of virus. All treated animals survived through the end of the study on day 18 while 60% of control animals died [50]. Additional studies in rhesus and cynomolgus macaques by the same group supported these findings (Table 1). Briefly, using a comparable dosing regimen, ribavirin resulted in 100% protection in the rhesus macaque study, with controls again experiencing 60% mortality [51]. In the cynomolgus macaque study, in which animals were treated with ribavirin intramuscularly with varying loading and maintenance doses and treatment initiated 0, 4, or 7 dpi, ribavirin treatment was 100% successful in preventing death when initiated within the first 4 days post-infection compared to a 93% mortality rate amongst untreated controls [52]. However, protection was reduced when treatment was delayed until 7 dpi, even when the maintenance and loading dose were increased, with delayed groups showing mortality rates ranging from 50–100% [52]. Delayed treatment groups did however show an increased time to death by 8+ days compared to untreated controls [52] (Table 1).

Although these initial ribavirin in vivo studies showed promising preclinical results, more recent studies in mice, guinea pigs, and cynomolgus macaques have not shown the same level of efficacy (Table 1 and Supplementary Table S3). A 2016 study using an IFNAR‒/‒ mouse model and a 80 mg/kg per day intraperitoneal dose initiated on day 0 or 4 post infection and continuing to day 11 showed no decrease in mortality rate in either group compared to controls, with only an increase in time to death in the day 0 animals [49]. Increasing the dose to 160 mg/kg per day with treatment starting on day 4 and continuing to day 11 resulted in a 20% survival rate and increased survival time [49]. Similarly, a Hartley guinea pig study showed no effect on overall survival but resulted in an increase in time to death in animals treated with 50 mg/kg/day of ribavirin starting on day 2 and continuing to day 14 (Supplementary Table S3) [24]. These discrepancies in efficacy could result from differences in the NHP and rodent models. However, it is presumably a more complex situation as a more recent cynomolgus macaque study showed that although time to death was increased by 10.5 days in animals who received a 30 mg/kg loading dose of ribavirin followed by a maintenance dose of 30 mg/kg/day (either given as a single dose or as 3 × 10 mg/kg doses every 8 h), all ribavirin treated animals still met euthanasia criteria by day 23 (Table 1) [53]. Although maintenance doses and time to first treatment in this study were similar to the original cynomolgus macaque study by Jahrling et al. 1984, the loading dose in the more recent study was reduced which may explain the differences in observed survival rates.

#### 3.1.3. Clinical Studies

Ribavirin is commonly used to treat LF as an off-label treatment option in humans, which is based primarily on results from a study published by McCormick et al. 1986. In this study, a significant drop in case fatality rate for LF patients with elevated aspartate aminotransferase (AST) levels was observed when treatment was initiated within 6 days after the onset of fever [36]. However, these results have recently been disputed due to several apparent limitations within the trial with additional data being released in March 2019 and a new analysis being performed by independent investigators [37,38,39]. The new analysis found that, in accordance with the original analysis, LF patients with high AST levels treated with ribavirin had reduced mortality rates (28.7%) compared to control groups (44.6%). However, surprisingly the new analysis also found that patients with normal AST levels had a higher rate of mortality when treated with ribavirin (9.1%) compared to control patients (4.1%) [38,39]. An additional 5 retroactive studies published between 2012 and 2018 testing ribavirin were compiled by Eberhardt et al. 2019 and included 372 participants from Sierra Leone or Nigeria [55,56,57,58,59]. The combined data demonstrated a reduced mortality rate of 38.2% in treated patients compared to 83% in control patients [38]. However, based on the retrospective determination of control groups common among all five studies, a risk for bias emerged based on the death of late presenting patients before ribavirin treatment was initiated, potentially resulting in an overestimation of the effect of ribavirin treatment on LF mortality [38].

Although evidence exists that ribavirin may have a beneficial effect on LF mortality rates in NHPs and humans, a randomized placebo controlled clinical trial demonstrating this efficacy is necessary before the usefulness of ribavirin against LF can be confidently determined. Additionally, the results from the re-analysis of the lone clinical trial underscores the need for caution when using ribavirin in a clinical setting as both a treatment of mild LF or as a post exposure prophylaxis. These results also emphasize that although ribavirin has a long history of use for LF, the extent of its efficacy is still unknown and alternative treatment options need to be pursued.

### 3.2. Favipiravir (T-705, 6-fluoro-3-hydroxypyrazine-2-carboxamide)

Favipiravir is an RdRp inhibitor that was first synthesized in 2000 through the modification of a pyrazine analog which had shown antiviral properties when screened against influenza virus (Supplementary Table S2) [60,61,62]. Since then, favipiravir has shown antiviral activity against multiple RNA viruses including orthomyxoviruses, arenaviruses, bunyaviruses, filoviruses, flaviviruses, and coronaviruses including SARS-CoV-2 [62,63,64,65,66]. In 2014, favipiravir was licensed in Japan for use against emergency influenza infections and stockpiles of the drug were established [67]. Favipiravir is also currently being investigated in multiple clinical trials for COVID-19 treatment [68].

#### 3.2.1. Mechanism of Action

Following administration and cellular uptake, favipiravir undergoes phosphoribosylation, resulting in the conversion of the favipiravir prodrug to favirpiravir-RTP (favipiravir ribofuranosyl-5′-triphosphate) which is responsible for antiviral activities [62,68,69]. Mechanistically, the drug mimics a purine nucleotide and is incorporated into viral RNA during replication or binds to conserved regions of the RdRp enzyme, resulting in the termination of viral transcription [62,68,70]. Similar to ribavirin, lethal mutagenesis (genome catastrophe) is a further potential mechanism, resulting in increased mutation frequencies, specifically for G to A and C to T transitions [71]. The broad-spectrum activity exerted by favipiravir against RNA viruses can be explained by the relatively conserved catalytic domain of the RdRp amongst several of these viruses [62].

#### 3.2.2. Preclinical Studies

Based on evidence that favipiravir protects against lethal challenge with other arenaviruses in hamster and guinea pigs [72,73,74] a 2015 study using the guinea pig-adapted LASV-Josiah virus in the Hartley guinea pig model established the efficacy of the drug against LASV infection (Supplementary Table S3) [24]. Two days following challenge with a lethal dose of LASV, guinea pigs were treated with favipiravir at 150 mg/kg/day or 300 mg/kg/day or with a ribavirin control at a dose of 50 mg/kg/day; a control group received only placebo treatments (Supplementary Table S3). While all placebo control and ribavirin treated animals met euthanasia criteria by day 13 and 30 respectively, the 300 mg/kg/day favipiravir treated group had a 100% survival rate throughout the duration of the study (42 days) with an associated 2–3 log infectious titer reduction compared to the placebo group. The 150 mg/kg/day favipiravir treated group had a significant increase in survival compared to the control and ribavirin groups but 2 of the 9 animals (22%) did meet euthanasia criteria by day 13. To investigate if delayed initiation of favipiravir treatment influenced the efficacy of the 300 mg/kg/day treatment regimen, groups of guinea pigs were challenged and treated starting 5, 7, and 9 days later. Delaying treatment to 5 and 7 days did not result in any mortality with all guinea pigs in both groups surviving. Delaying treatment to 9 days resulted in 1 of 6 guinea pigs (17%) being euthanized on day 14 [24]. In the placebo and day 7 and 9 delayed treatment groups, fever was observed by 7 dpi. In both treatment groups fevers began to subside starting the day after treatment initiation with 67% of day 7 treated animals having normal temperatures within 2 days (2 guinea pigs resolved fever after 4 and 8 days) while all of the day 9 treated animals had resolved fevers within 2 to 4 days after treatments were initiated [24].

Further assessment using a lethal IFNAR‒/‒ LF mouse model with dosing regimens of 75 mg/kg/day or 150 mg/kg/day initiated 4 dpi did suppress viremia by 2 logs between days 4 and 8 but the treatments did not result in significant improvements in survival or time to death compared to the placebo treated group [49]. However, increasing the dose to 300 mg/kg/day did result in 100% survival and an associated decrease in viremia and viral titers in the organs compared to the placebo group (Supplementary Table S3). In comparison to the previously discussed high dose ribavirin group (80 mg/kg/day initiated on day 4) (see above), which resulted in only 20% survival, favipiravir shows a clear survival benefit [49].

Administration of favipiravir in NHP studies have also resulted in an increase in survival (Table 1). A 2018 study tested the efficacy of 300 mg/kg intravenous loading dose administered 4 dpi with subsequent 300 mg/kg subcutaneous drug treatments every 24 h for 13 days in cynomolgus macaques [54]. By 6 dpi clinical scores for 75% of treated animals plateaued and all of the treated animals survived throughout the study period (56 days). In contrast, control animals experienced a substantial increase in observable disease by 8 dpi and all animals reached euthanasia criteria by 12 dpi [54]. In an associated cynomolgus macaque study, a reduced favipiravir dose of 300 mg/kg intravenous loading dose administered 4 dpi with subsequent 50 mg/kg subcutaneous treatments every 8 h resulted in no significant benefit on survival compared to untreated controls [53]. Of note, in these studies the high dose favipiravir resulted in 100% survival compared to ribavirin which, although an increased time to euthanasia was observed, resulted in no survival.

#### 3.2.3. Clinical Studies

To this point no clinical trials assessing the efficacy of favipiravir against LASV have been conducted. A combination of ribavirin and favipiravir was successfully used to treat two LF cases in 2017 with both patients surviving [75].

Favipiravir has shown promising results in animal studies against LASV infection. Notably, in all reported animal studies favipiravir outperformed ribavirin treatments or controls based on survival and time to death. These results emphasize a critical need for clinical trials assessing the efficacy of favipiravir in comparison to ribavirin or in combination with ribavirin or other treatment options against LASV infection.

### 3.3. Stampidine (methyl 2-[[(4-bromophenoxy)-[[(2S,5R)-5-(5-methyl-2,4-dioxopyrimidin-1-yl)-2,5-dihydrofuran-2-yl]methoxy]phosphoryl]amino]propanoate)

Stampidine is a nucleoside analog derived from d4T, a potent retroviral reverse transcriptase inhibitor (Supplementary Table S2) [76,77]. Using a semi lethal CBA mouse model, stampidine shows increased survival against LASV challenge at two dose regimens compared to vehicle controls [77] (Supplemental Table S3). Mice were treated with 25 mg/kg or 50 mg/kg 24 and 1 h before and 24, 48, 72, and 96 h after challenge with 1000 pfu of LASV Josiah. While only 28% of the animals in the vehicle control group survived, 90% and 75% of the animals in the 50 mg/kg and 25 mg/kg treatments groups survived, respectively [77]. These results warrant further investigation in animal models.

### 3.4. ST-193 (1-(4-methoxyphenyl)-N-[(4-propan-2-ylphenyl)methyl]benzimidazol-5-amine)

ST-193 is a small molecule viral entry inhibitor of LASV (Supplementary Table S2). Its parent compound ST-37 was initially identified in a high throughput screen using a virus pseudotype with the LASV glycoprotein [78]. Analysis of ST-37 analogs revealed that ST-193 had significantly more potent antiviral activity with an IC_50_ of 1.6 nM. LHF-535 is a chemical analog of ST-193 which has also shown inhibition in the sub-nanomolar IC_50_ range [79]. To date, there is only a single published study testing the efficacy of ST-193 in vivo [80]. Using the lethal Hartley guinea pig model, animals were treated intraperitoneally 1 h prior to infection with either 25 mg/kg per day or 80 mg/kg per day of ST-193 up to day 14 (Supplemental Table S3). As controls, ribavirin at 25 mg/kg per day or vehicle were also incorporated in the study. Guinea pigs were challenged with a lethal dose of LASV Josiah and followed for 30 days. Consistent with earlier studies, vehicle and ribavirin treated animals all succumbed to disease within the study period with ribavirin treated animals having an increased time to death compared to the vehicle group. Both ST-193 treatment groups had 63% survival throughout the study period, showing a benefit in survival over the control and ribavirin groups [80]. Additional animal studies are suggested to further analyze this potential treatment.

### 3.5. Immune Plasma

The practice of using passive immune therapy to treat viral infections has a long history, with the Spanish influenza (1918–1920) being the first viral infection for which clinical trials showed efficacy [81,82,83]. Since then, convalescent plasma therapy has been considered as a treatment option for multiple other viruses including Ebola virus and most recently SARS-CoV-2 [81,84,85]. Of note, convalescent plasma is the only approved treatment for Argentine hemorrhagic fever which is caused by, Junin virus, a New World mammarenavirus [86]. Use of convalescent plasma treatment in human LF cases preceded animal studies and was first used to treat a virologist in Connecticut shortly after the disease was first discovered in 1969. Convalescent plasma therapy has thereafter been used in a number of LF patient settings [87,88,89,90,91,92].

#### 3.5.1. Preclinical Studies

LASV immune plasma treatments have been extensively tested in strain 13 guinea pigs and a number of cynomolgus macaque studies [52,93,94]. The study parameters and specific results from these studies are summarized in Table 2 and Supplementary Table S4.

Various dose regimens, antibody titers, and antibody sources were tested using the lethal strain 13 guinea pig model (Supplementary Table S4) [93]. Plasma was derived from 32, 45, 60, 90, and 180 day convalescent Hartley guinea pigs, 180–240 day convalescent rhesus macaques, and a 2–3 year convalescent human infected in Liberia. Treatment with various pools and different titrations of convalescent plasma from Hartley guinea pigs, resulted in high levels of protection in strain 13 guinea pigs, with 80–100% survival compared to 0% in control groups. Concentration of LASV neutralizing antibodies within the plasma appeared important, as high protection was observed for intraperitoneal doses of 6 mL/kg or 12 mL/kg starting on day 0 and repeated on days 3 and 6 if the plasma pool had a calculated Log10 neutralization index (LNI) of 2.0 or higher [93]. Plasma treatments below 6 mL/kg and 2.0 LNI resulted in partial or absent protection. Interestingly, Hartley guinea pigs recovering from LASV infection did not develop the required 2.0 LNI until at least 60 days post infection. Treatment of strain 13 guinea pigs with convalescent rhesus macaque plasma, resulted in complete protection when LNI levels were above 2.5 and 6 mL/kg of plasma was administered. Similar results were observed when guinea pigs were treated with human plasma from the convalescent Liberian LF patient. Greater LNI and protective efficacy was observed when the guinea pigs were challenged with the Liberian strain Z-132 as opposed to Josiah strain from Sierra Leone [93]. Although both Z-132 and Josiah are clade IV strains, they are serologically distinct and these differences indicate that immune plasma treatments may need to be administered at higher levels across viral strains to achieve protection.

Protective efficacy of immune plasma treatment from monkey and human sources has also been investigated in the cynomolgus macaque model (Table 2) [52,94]. Rhesus macaque and human sera were sourced from the same survivors referenced above. Treatment of cynomolgus macaques with 1 mL/kg of 4.1 LNI immune plasma on days 0, 3, and 6 or days 4, 7, and 10 after infection resulted in survival rates of 88% and 66%, respectively, compared to 7% in untreated controls [52]. When treatment was delayed until day 7, 10, and 13 after infection, survival rates dropped to 17% [52]. Similar to guinea pigs [93], cynomolgus macaques were completely protected from lethal LASV infection if monkey immune plasma was given at a minimum dose of 3 mg/kg with an LNI of 2.6 or higher compared to untreated controls with only 5% survival [94]. When macaques were treated with undiluted immune plasma from the recovered Liberian LF patient, treatment was noted to be ineffective when the Sierra Leone Josiah strain was used (0% survival) for challenge compared to the Liberian/Guinean Macenta strain (75–100% survival), further emphasizing the importance of strain specificity for immune plasma treatment [94].

Taken together, these animal studies indicate that immune plasma therapy may be a viable treatment option for LASV infections if treatment is initiated quickly after onset of symptoms, the immune plasma has high levels of LASV neutralizing antibodies, and strain specificity is taken into account.

#### 3.5.2. Clinical Studies

Although convalescent plasma treatment has been used in multiple patient settings with mixed success [87,88,89,90,91,92], only a single clinical trial has been conducted [36]. In stage I of the trial, 31 patients were treated with 1 unit of LF convalescent plasma which equated to about 4 mL/kg while an additional group of 22 patients was treated with 2 units (8 mL/kg). These two treatment regimens were subsequently combined into 1 plasma treatment group for statistical analysis. The convalescent plasma had an immunofluorescent-antibody titer of at least 1 to 128 and treatment was initiated within 24 h post admission. In stage II, patients were admitted into the study based on a LASV diagnosis and AST levels above 150 IU per liter. In stage II, 1 unit of plasma treatment was given in combination with ribavirin treatment to 33 patients. For stage I, the immune plasma treatment showed no significant decrease in mortality rate compared to untreated controls and performed worse than ribavirin in all subgroup analysis based on specific infection markers such as AST levels and viremia [36]. In stage II, adding immune plasma treatments to ribavirin treatment resulted in no increases in survival.

Although the results from the animal studies indicated that convalescent plasma therapy has potential as a treatment for LASV, the sole clinical study showed no efficacy. The animal studies emphasized the importance of neutralization index, rapid treatment initiation, and the importance of strain specificity; notably, these factors were not considered in the clinical trial. While time to treatment initiation is difficult to control based on symptomology and patient admittance into the healthcare setting, convalescent plasma could be sourced from relevant patient populations and concentrated to reach necessary neutralization indexes which may yield more beneficial treatment outcomes [91]. Although convalescent plasma treatment has potential to be an effective treatment for LF, research into the use of more specific and targeted monoclonal antibodies is also indicated.

### 3.6. Monoclonal Antibodies

Monoclonal antibody (MAb) treatments have been developed for multiple diseases and several viruses with Synagis, a MAb treatment for respiratory syncytial virus infection, being the first virus targeting MAb treatment approved by the FDA [16,96]. In 2020, Inmazeb, a cocktail of three monoclonal antibodies was approved by the FDA for use against Ebola virus [97]. Due to technological advances, including high specificity and targeting of specific single epitopes, MAb humanization, predictable consistency in manufacturing, and increased safety compared to polyclonal antibody/convalescent plasma treatments, MAbs have high potential for the treatment of virus caused illnesses, including LF [98,99].

In 2016, 113 human MAbs (huMAbs) targeting LASV glycoproteins were identified from memory B cells of LF survivors [100]. Of these identified MAbs, 16 were found to be neutralizing in vitro in LASV pseudotype and LASV plaque reduction assays. Thirteen of the neutralizing antibodies bound to the assembled glycoprotein complex while the remaining 3 bound to GP1 only [100]. Three of these MAbs (8.9F, 12.1F, and 37.2D) showed activity across LASV Clades II, III, and IV [101].

#### 3.6.1. Preclinical Studies

Eleven of the 13 neutralizing antibodies described by Robinson et al. 2016 were tested in challenge studies in Hartley guinea pigs (Supplementary Table S4) [101]. Groups of animals were treated intraperitoneally with 30 mg/kg of the huMAbs following challenge on day 0 and were given subsequent treatments on 3 and 6 dpi. While controls animals, which were either left untreated or were treated with a huMAb control, had a pooled survival of only 6%, complete and partial protection was observed in the huMAb treatment groups. Specifically, huMAbs 37.7H, 12.1F, 2.9D, 25.6A, and 8.9F treatment resulted in complete survival, while huMAbs 37.2D and 19.7E conferred 90% protection (studies were repeated and pooled results were reported). HuMAb 37.2G protected 80% of challenged animals. HuMAbs 36.1F, 25.10C, and 10.4B performed less impressively with survival rates ranging between 20–40% [101]. Based on these data, huMAbs 37.7H, 37.2D, 12.1F, 8.9F and 19.7E were subsequently tested in the cynomolgus macaque model (Table 2) [95]. Treatment regimens of 15 mg/kg administered intravenously at 0, 4, and 8 dpi resulted in 100% survival for huMAbs 37.2D, 12.1F, 8.9F, and 37.7H compared to controls (N = 7) with 0% survival. HuMAb 19.7E, when treatment was initiated on day 0 and repeated on day 5, had 75% survival [95]. Combining huMAbs 8.9F, 12.1F, and 37.2D at 15 mg/kg each with treatments 3 days apart, protected all NHPs even when treatment initiation was delayed until 8 dpi [95].

#### 3.6.2. Clinical Studies

No human studies using MAbs for LF treatment have been conducted. Given the positive preclinical results, clinical trials assessing their efficacy in humans are needed. However, huMAbs treatments are expensive and technological and manufacturing advances need to be made for treatment to be broadly available to the low-income communities that are most affected by LASV.

## 4. Discussion

LASV was first described in 1969 and although over 50 years have passed, no treatment has thus far been approved. The burden of LASV on much of West Africa combined with its history of nosocomial human to human transmission events, and potential for transmission to non-endemic countries make it critical that viable treatments be developed to control and prevent LASV outbreaks. This need is underscored by LASV’s designation as a priority pathogen by the WHO in 2018. Supportive treatment including fluid replacement, electrolyte balancing, and oxygen supplementation as well as dialysis, when indicated, are the primary medical interventions for LF cases [7,16,17]. Additionally, ribavirin has been used as an off-label treatment option for LF based on a single clinical trial supporting its efficacy [36]. However, recent re-analysis of results from this study call into question some of the findings [37,38] and the use of ribavirin for LF should be reevaluated. Furthermore, no LASV vaccine has moved beyond the preclinical stage and shown safety or efficacy in humans [102]. Rodent control interventions have shown some success in reducing the abundance of Mastomys (the natural reservoir of LASV) in village settings, but numbers rebound shortly after interventions cease and such interventions can be cost and labor intensive in already impoverished communities [103]. Together, the lack of treatments, vaccines, and rodent control strategies leaves infectious disease and public health responses with extremely limited options for preventing and treating LF.

In this review, we have discussed the major LF antiviral options currently in development and compiled the details of their corresponding preclinical and clinical studies (Table 1 and Table 2 and Supplementary Tables S3 and S4). Favipiravir and huMAbs are most promising and likely should replace ribavirin as first choice until efficacy of ribavirin is reevaluated. Favipiravir has shown efficacy in mice, guinea pigs, and NHPs, outperforming ribavirin in all comparative published studies [49,53,54,103]. It has also been proven safe for use against emergency influenza virus with licensure in Japan [67]. Therefore, favipiravir should be urgently moved into clinical trials either as a mono- or combined therapy. Comparison with ribavirin monotherapy would be of scientific interest but seems questionable based on recent efficacy data.

Combination drug therapy is common in other virus infections such as HIV/AIDS and hepatitis C virus and should also be considered for LF. Specifically, treatment combinations that target distinct viral mechanisms should be emphasized and could function to increase overall treatment efficacy and avert the potential development of antiviral resistance by LASV against any one drug. To determine the optimal drug combinations, further mechanistic and preclinical efficacy studies should be performed on promising drug candidates. Combination therapy of favipiravir, which is believed to target the RdRp enzyme [62,68,70], and ribavirin, with multiple proposed mechanisms including IMPDH inhibition [42,43,44,45], could be considered as they have shown synergistic effects in a LASV rodent model [49]. Stampidine, characterized as a retroviral reverse transcriptase inhibitor [76,77], and ST-193, a viral entry inhibitor of LASV [78], also have mechanisms that would be amendable for combination therapy with one another or with ribavirin and favipiravir. Additionally, glycoprotein targeting huMAbs are strong candidates for both individual and combined therapy.

HuMAb therapy for LF has shown astonishing efficacy in preclinical models. Specifically, the cocktail of huMAbs 8.9F + 12.1F + 37.2D, which provided 100% protection against lethal LASV challenge in cynomolgus macaques even when treatment was delayed until 8 dpi [95], should be considered for clinical trials. A drawback of MAbs are their high specificity with treatment cocktails potentially having to be clade- or even strain-adapted; small drug molecules interfering with the replicase complex likely show a broader efficacy. In addition, huMAb treatments will likely continue to be cost prohibitive for those countries where LASV exerts it greatest burden, highlighting the need for research to reduce the cost of producing huMAb treatments and making them broadly available. Alternatively, combined therapy of favipiravir and immune plasma could be considered due to the protection observed in a previous study in which cynomolgus macaques were treated with a combination of ribavirin and immune plasma [52].

In clinical settings, LF is often only considered after other diagnoses such as typhoid and malaria have been ruled out. The importance of initiating LF treatment early was heavily reinforced in the reviewed preclinical studies. These findings emphasize the need for diagnostic infrastructure to rapidly and accurately diagnose LASV infections and allow for the initiation of specific treatments as early as possible.

The current COVID-19 pandemic has emphasized the need for preemptive efforts to establish countermeasures for emerging infectious diseases. LASV, having been notorious for importation through infected individuals, needs to be considered as a pathogen of high priority for future clinical investigation.

## Figures and Tables

**Table 1:** Pharmaceutical based therapeutics for Lassa virus (LASV) in non-human primates (NHP) models.

Animal Model	Challenge	Treatment Regimen	Survival	Reference
**Ribavirin**				
Rhesus Macaque	SQ 10,000 PFU LASV	(1) Loading dose 50 mg/kg 0 dpi + 10 mg/kg three times daily to day 18 IM (N = 4)	(1) 100%	[50]
		(2) Loading dose 5 dpi + 10 mg/kg three times daily to day 18 IM (N = 4)	(2) 100%	
		(3) Controls (N = 10)	(3) 60%	
Rhesus Macaque	SQ 1.2 × 10^6^ PFU LASV Josiah	(1) Loading dose 50 mg/kg 0 dpi + 10 mg/kg three times daily to day 18 SQ (N = 4)	(1) 100%	[51]
		(2) Loading dose 50 mg/kg 5 dpi + 10 mg/kg three times daily to day 18 SQ (N = 4)	(2) 100%	
		(3) Controls (N = 1(0)	(3) 60%	
Cynomolgus Macaque	SQ 1.2 × 10^6^ PFU LASV Josiah	(1) Loading dose 150 mg/kg 0 dpi + 15 mg/kg twice daily to day 18 IM (N = 4)	(1) 100%	[52]
		(2) Loading dose 150 mg/kg 4 dpi + 15 mg/kg twice daily to day 18 IM (N = 4)	(2) 100%	
		(3) Loading dose 150 mg/kg 7 dpi + 15 mg/kg twice daily to day 18 IM (N = 8)	(3) 50%	
		(4) Loading dose 300 mg/kg 7 dpi + 30 mg/kg twice daily to day 18 IM (N = 4)	(4) 25%	
		(5) Loading dose 450 mg/kg 7 dpi + 45 mg/kg twice daily to day 18 IM (N = 6)	(5) 0%	
		(6) Untreated controls (N = 1(4)	(6) 0%	
Cynomolgus Macaque	IM 1 × 10^4^ TCID50 LASV Josiah	(1) 30 mg/kg loading dose 4 dpi + 10 mg/kg every 8 h SQ (N = 4)	(1) 0%	[53,54]
		(2) 30 mg/kg loading dose + 30 mg/kg once daily SQ	(2) 0%	
		(3) Placebo controls	(3) 0%	
**Favipiravir**				
Cynomolgus Macaque	IM 1 × 10^4^ TCID50 LASV Josiah	(1) 300 mg/kg IV loading dose 4 dpi + 300 mg/kg per day SQ treatments for 13 more days (N = 4)	(1) 100%	[53,54]
		(2) Placebo treated starting 4 dpi to death (N = 4)	(2) 0%	
Cynomolgus Macaque	IM 1 × 10^4^ TCID50 LASV Josiah	(1) 300 mg/kg IV loading dose 4 dpi + 50 mg/kg every 8 h SQ treatments for 13 more days (N = 4)	(1) 0%	[53,54]
		(2) Placebo treated 4 dpi to death (N = 8)	(2) 0%	

IM = intramuscular. SQ = subcutaneous. DPI = days post infection. N = group size. PFU = plaque forming units. FFU = fluorescent focus units. GPA = guinea pig adapted. LASV = Lassa virus.

**Table 2:** Antibody based therapeutics for LASV in NHP models.

Animal Model	Challenge	Treatment Regimen	Survival	Reference
**Immune Plasma**				
Cynomolgus Macaque	SQ 1.0 × 10^6.1^ PFU LASV Josiah	Plasma from Rhesus Macaque convalescent 180–240 days		[94]
		(1) 1 mL/kg LNI 4.1, IFA 1280 plasma given 0, 3, and 6 dpi IV (N = 8)	(1) 88%	
		(2) 1 mL/kg LNI 2.6, IFA 320 plasma given 0, 3, and 6 dpi IV (N = 3)	(2) 0%	
		(3) 3 mL/kg LNI 2.6, IFA 320 plasma given 0, 3, and 6 dpi IV (N = 4)	(3) 100%	
		(4) 3 mL/kg LNI 1.5, IFA 80 plasma given 0, 3, and 6 dpi IV (N = 3)	(4) 0%	
		(5) 3 mL/kg LNI 1.5, IFA 80 plasma given 0, 3, 6, 9, and 12 dpi IV (N = 3)	(5) 0%	
		(6) 3 mL/kg LNI 0.5, IFA 20 plasma given 0, 3, and 6 dpi IV (N = 3)	(6) 0%	
		(7) Untreated controls (N = 20)	(7) 5%	
Cynomolgus Macaque	SQ 1.0 × 10^6.1^ PFU LASV Josiah	Plasma from human 2–3 years convalescent		[94]
		(1) 3 mL/kg LNI 1.6 plasma given 0, 3, and 6 dpi IV (N = 4)	(1) 0%	
		(2) 12 mL/kg LNI 1.6 plasm given 0, 3, and 6 dpi IV (N = 4)	(2) 0%	
		(3) Control plasma (N = 4)	(3) 0%	
Cynomolgus Macaque	SQ 1.0 × 10^6^ PFU LASV Macenta	Plasma from human 2–3 years convalescent		[94]
		(1) 3 mL/kg LNI 2.8 plasma given 0, 3, and 6 dpi IV (N = 4)	(1) 75%	
		(2) 12 mL/kg LNI 2.8 plasm given 0, 3, and 6 dpi IV (N = 4)	(2) 100%	
		(3) Untreated controls (N = 4)	(3) 0%	
Cynomolgus Macaque	SQ 1.0 × 10^6.1^ PFU LASV Josiah	Plasma from Rhesus Macaque convalescent 180–240 days		[52]
		(1) 1 mL/kg 4.1 LNI plasma given 0, 3, and 6 dpi IV (N = 8)	(1) 88%	
		(2) 1 mL/kg 4.1 LNI plasma given 4, 7, and 10 dpi IV (N = 3)	(2) 66%	
		(3) 1 mL/kg 4.1 LNI plasma given 7, 10, and 13 dpi IV (N = 6)	(3) 17%	
		(4) Untreated controls (N = 14)	(4) 7%	
**HuMAb 8.9F**				
Cynomolgus Macaque	IM 3500 PFU target dose LASV Josiah	(1) 15 mg/kg on 0, 4, and 8 dpi (N = 4)	(1) 100%	[95]
		(2) Pooled controls (N = 7)	(2) 0%	
**HuMAb 12.1F**				
Cynomolgus Macaque	IM 3500 PFU target dose LASV Josiah	(1) 15 mg/kg on 0, 4 and 8 dpi (N = 3)	(1) 100%	[95]
		(2) Pooled controls (N = 7)	(2) 0%	
**HuMAb 37.7H**				
Cynomolgus Macaque	IM 3500 PFU target dose LASV Josiah	(1) 15 mg/kg on 0, 4, and 8 dpi (N = 3)	(1) 100%	[95]
		(2) Pooled controls (N = 7)	(2) 0%	
**HuMAb 37.2D**				
Cynomolgus Macaque	IM 3500 PFU target dose LASV Josiah	(1) 15 mg/kg on 0, 4 and 8 dpi (N = 4)	(1) 100%	[95]
		(2) 6 mg/kg on 0, 4, and 8 dpi IV (N = 2)	(2) 100%	
		(3) Pooled controls (N = 7)	(3) 0%	
**HuMAb 19.7E**				
Cynomolgus Macaque	IM 3500 PFU target dose LASV Josiah	(1) 15 mg/kg on 0 and 5 dpi IV (N = 4)	(1) 75%	[95]
		(2) Pooled controls (N = 7)	(2) 0%	
**HuMAb 19.7E + 37.2D**				
Cynomolgus Macaque	IM 3500 PFU target dose LASV Josiah	(1) Total dose 15 mg/kg on 0, 4, and 8 dpi IV (N = 4)	(1) 100%	[95]
		Equal mixture		
		(2) Pooled controls (N = 6)	(2) 0%	
**HuMAb 8.9F + 12.1F + 37.2D**				
Cynomolgus Macaque	IM 3500 PFU target dose LASV Josiah	(1) 15 mg/kg on 3, 6, and 9 dpi IV (N = 4)	(1) 100%	[95]
		(2) 15 mg/kg on 6, 9, and 12 dpi IV (N = 4)	(2) 100%	
		(3) 15 mg/kg on 8, 11, and 14 dpi IV (N = 5)	(3) 100%	
		(4) Pooled controls (N = 6)	(4) 0%	
		15 mg/kg of each MAb		

IM = intramuscular. SQ = subcutaneous. LNI = Log10 neutralization index = (Log10 (PFU in control)-Log10 (PFU in test serum)) (LNI determined based on challenge strain) (Jahrling 1983). IFA = indirect fluorescent antibody. IP = intraperitoneal. IV = intravenous. DPI = days post infection. N = group size. PFU = plaque forming units. LASV = Lassa virus.

## Data Availability

No new data were created or analyzed in this study. Data sharing is not applicable to this article.

## Supplementary Materials

The following are available online at https://www.mdpi.com/article/10.3390/microorganisms9040772/s1, Table S1: Abbreviation Key, Table S2: Chemical Structures, Table S3: Therapeutics for LASV in rodent animal models, Table S4: Antibody based therapeutics for LASV in rodent animal models.
