# Supplementary material for: Lassa Virus Treatment Options
Source: Microorganisms. 2021 Apr 7;9(4):772. doi: 10.3390/microorganisms9040772 (PMC8067676; doi:10.3390/microorganisms9040772)
Supplement: Supplementary file 1 [file microorganisms-09-00772-s001.pdf]

Supplementary Table S1: Abbreviation key

Abbreviation	Definition
LASV	Lassa virus
LF	Lassa Fever
RdRp	RNA-dependent RNA polymerase
GPC	Glycoprotein precursor complex
GP	Glycoprotein
Mastomys	*Mastomys natalensis*
NHP	Non-human primate
RMP	Ribavirin monophosphate
IMPDH	Inosine monophosphate dehydrogenase
GTP	Guanosine triphosphate
RTP	Ribavirin triphosphate
AST	Aspartate aminotransferase
LNI	Log10 neutralization index
MAb	Monoclonal antibody
huMAb	Human monoclonal antibody

Supplementary Table S2: Chemical Structures

Ribavirin	Favipiravir
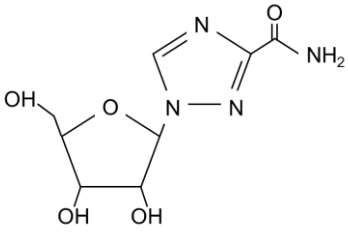 MW: 244.2 g/mol	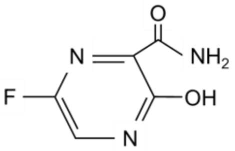 MW: 157.1 g/mol
**Stampidine**	**ST-193**
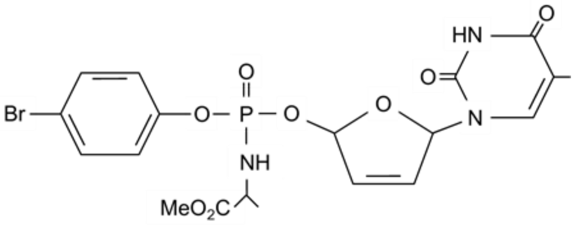 MW: 544.3 g/mol	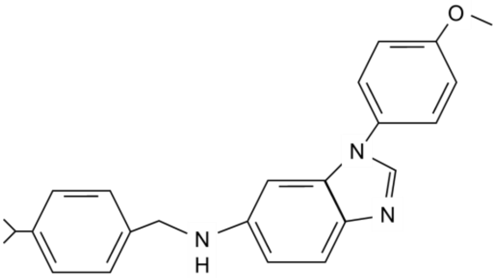 MW: 371.5 g/mol

Supplementary Table S3. Therapeutics for LASV in rodent animal models.

Animal Model	Challenge	Treatment Regimen	Survival	Reference
		**Ribavirin**		
Ifnar-/-B6 mouse	IP 1,000 FFU LASV Ba366	1) 80 mg/kg per day IP starting 0 dpi to death (N=5)<br>2) 80 mg/kg per day IP starting 4 dpi to death (N=5)<br>3) 80 mg/kg twice per day IP starting 4 dpi to 11 (N=5)<br>4) Placebo treated starting 4 dpi to death (N=3)	1) 0%<br>2) 0%<br>3) 20%<br>4) 0%	[49]
Hartley Guinea Pig	IP 1x10^5 TCID50 GPA-LASV	1) 50 mg/kg per day SQ starting 2 dpi to 14 dpi (N=9)<br>2) Placebo treated starting 2 dpi to death (N=9)	1) 0%<br>2) 0%	[53]
		**Favipiravir**		
Hartley Guinea Pig	IP 1x10^5 TCID50 GPA-LASV	1) 150 mg/kg per day SQ starting 2 dpi to 14 dpi (N=9)<br>2) 300 mg/kg per day SQ starting 2 dpi to 14 dpi (N=9)<br>3) Placebo treated starting 2 dpi to death (N=9)	1) 78%<br>2) 100%<br>3) 0%	[53]
Hartley Guinea Pig	IP 1x10^5 TCID50 GPA-LASV	1) 300 mg/kg per day SQ starting 5 dpi (N=6)<br>2) 300 mg/kg per day SQ starting 7 dpi (N=6)<br>3) 300 mg/kg per day SQ starting 9 dpi (N=6)<br>4) Placebo treated starting 5 dpi (N=6)	1) 100%<br>2) 100%<br>3) 83%<br>4) 0%	[53]
IFNAR-/-B6 mouse	IP 1,000 FFU LASV Ba366	1) 75 mg/kg per day oral starting 4 dpi to death (N=5)<br>2) 150 mg/kg per day oral starting 4 dpi to death (N=5)<br>3) 300 mg/kg per day oral starting 4 dpi to 11 dpi (N=5)<br>4) Placebo treated group starting 4 dpi to 9 dpi (N=7)	1) 0%<br>2) 0%<br>3) 100%<br>4) 0%	[49]
		**ST-193**		
Hartley Guinea Pig	SQ 1,000 pfu LASV Josiah	1) 25 mg/kg per day IP starting 1 h pre infection to day 14 (N=8)<br>2) 80 mg/kg per day IP starting 1 h pre infection to day 14 (N=8)<br>3) Ribavirin control: 25 mg/kg per day IP starting 1 h preinfection to day 14 (N=8)<br>4) Vehicle control starting 1 h preinfecton to day 14 (N=7)	1) 63%<br>2) 63%<br>3) 0%<br>4) 0%	[81]
		**Stampidine**		
CBA mouse	IC 1,000 pfu LASV Josiah	1) 25 mg/kg per day IP starting 24 h pre infection + 1 h preinfection + every subsequent 24 h until 96 h (N=8)<br>2) 50 mg/kg per day IP starting 24 h pre infection + 1 h preinfection + every subsequent 24 h until 96 h (N = 10)<br>3) Vehicle treatment with same regimen (N=18)	1) 75%<br>2) 90%<br>3) 28%	[78]

IP = Intraperitoneal. SQ = Subcutaneous. IC = Intracerebral DPI = Days post infection. N = group size. PFU = plaque forming units. FFU = Fluorescent focus units. GPA = Guinea pig adapted. LASV = Lassa virus.

Supplementary Table S4. Antibody based therapeutics for LASV in rodent animal models.

Animal Model	Challenge	Treatment Regimen	Survival	Reference
		**Immune Plasma**		
Strain 13 Guinea Pig	SQ 1x10^3.4 PFU LASV Josiah	Plasma from Hartley guinea pigs 32 days convalescent<br>1) 6 mL/kg of LNI .3, IFA 2,560 plasma given 0, 3, and 6 dpi IP (N = 10)<br>2) 6 mL/kg of LNI .1, IFA 640 plasma given 0, 3, and 6 dpi (N = 10)<br>3) 6 mL/kg of LNI .1, IFA 160 plasma given 0, 3,and 6 dpi (N = 10)<br>4) Control plasma (N = 10)	1) 0%<br>2) 0%<br>3) 0%<br>4) 0%	[94]
Strain 13 Guinea Pig	SQ 1x10^3.4 PFU LASV Josiah	Plasma from Hartley guinea pigs 45 days convalescent<br>1) 6 mL/kg of LNI .9, IFA 2,560 plasma given 0, 3, and 6 dpi<br>2) 6 mL/kg of LNI .6, IFA 320 plasma given 0, 3, and 6 dpi (N = 10)<br>3) 6 mL/kg of LNI .3, IFA 160 plasma given 0, 3,and 6 dpi (N = 10)<br>4) Control plasma (N = 10)	1) 0%<br>2) 0%<br>3) 0%<br>4) 0%	[94]
Strain 13 Guinea Pig	SQ 1x10^3.4 PFU LASV Josiah	Plasma from Hartley guinea pigs 90 days convalescent<br>1) 6 mL/kg of LNI 3.4, IFA 2,560 plasma given 0, 3, and 6 dpi<br>2) 6 mL/kg of LNI 3.1, IFA 640 plasma given 0, 3, and 6 dpi (N = 10)<br>3) 6 mL/kg of LNI 2.0, IFA 160 plasma given 0, 3,and 6 dpi (N = 10)<br>4) 6 mL/kg of LNI 1.4, IFA 40 plasma given 0, 3,and 6 dpi (N = 10)<br>5) Control plasma (N = 10)	1) 100%<br>2) 100%<br>3) 90%<br>4) 50%<br>5) 0%	[94]
Strain 13 Guinea Pig	SQ 1x10^3.4 PFU LASV Josiah	Plasma from Hartley guinea pigs 180 days convalescent<br>1) 6 mL/kg of LNI 3.8, IFA 2,560 plasma given 0, 3, and 6 dpi<br>2) 6 mL/kg of LNI 2.8, IFA 640 plasma given 0, 3, and 6 dpi (N = 10)<br>3) 6 mL/kg of LNI 2.2, IFA 320 plasma given 0, 3,and 6 dpi (N = 10)<br>4) 6 mL/kg of LNI 1.5, IFA 40 plasma given 0, 3,and 6 dpi (N = 10)<br>5) 6 mL/kg of LNI 0.8, IFA 10 plasma given 0, 3, and 6 dpi (N = 10)<br>6) Control plasma (N = 10)	1) 100%<br>2) 100%<br>3) 80%<br>4) 30%<br>5) 0%<br>6) 0%	[94]
Strain 13 Guinea Pig	SQ 1x10^3.4 PFU LASV Josiah	Plasma from Hartley guinea pigs 60 days convalescent<br>1) 1 mL/kg LNI 2.2 plasma given 0, 3, and 6 dpi (N = 10)<br>2) 3 mL/kg LNI 2.2 plasma given 0, 3, and 6 dpi (N = 10)<br>3) 6 mL/kg LNI 2.2 plasma given 0, 3, and 6 dpi (N = 10)<br>4) 12 mL/kg LNI 2.2 plasma given 0, 3, and 6 dpi (N = 10)<br>5) Untreated control (N = 10)	1) 0%<br>2) 50%<br>3) 100%<br>4) 100%<br>5) 0%	[94]
Strain 13 Guinea Pig	SQ 1x10^3.4 PFU LASV Josiah	Plasma from Hartley guinea pigs 180 days convalescent<br>1) 1 mL/kg LNI 2.0 plasma given 0, 3, and 6 dpi (N = 10)<br>2) 3 mL/kg LNI 2.0 plasma given 0, 3, and 6 dpi (N = 10)<br>3) 6 mL/kg LNI 2.0 plasma given 0, 3, and 6 dpi (N = 10)<br>4) 12 mL/kg LNI 2.0 plasma given 0, 3, and 6 dpi (N = 10)<br>5) Untreated control (N = 10)	1) 10%<br>2) 60%<br>3) 100%<br>4) 100%<br>5) 0%	[94]
Strain 13 Guinea Pig	SQ 1x10^4 PFU LASV Josiah	Plasma from Rhesus Macaque convalescent 180-240 days<br>1) 6 mL/kg LNI 3.6 plasma given 0, 3, and 6 dpi (N=5)<br>2) 1) 6 mL/kg LNI 2.5 plasma given 0, 3, and 6 dpi (N=5)<br>3) 6 mL/kg LNI 1.2 plasma given 0, 3, and 6 dpi (N=5)<br>4) 6 mL/kg LNI 0.5 plasma given 0, 3, and 6 dpi (N=5)<br>5) Control plasma (N=5)	1) 100%<br>2) 100%<br>3) 0%<br>4) 0%<br>5) 0%	[94]
Strain 13 Guinea Pig	SQ 1x10^4 PFU LASV Josiah	Plasma from human 2-3 years convalescent<br>1) 3 mL/kg LNI 1.6 plasma given 0, 3, and 6 dpi (N = 10)<br>2) 6 mL/kg LNI 1.6 plasma given 0, 3, and 6 dpi (N = 10)<br>3) 12 mL/kg LNI 1.6 plasma given 0, 3, and 6 dpi (N = 10)<br>4) No treatment (N = 10)	1) 0%<br>2) 50%<br>3) 100%<br>4) 0%	[94]
Strain 13 Guinea Pig	SQ 1x10^4.3 PFU LASV Z-132	Plasma from human 2-3 years convalescent<br>1) 3 mL/kg LNI 2.8 plasma given 0, 3, and 6 dpi (N = 10)<br>2) 6 mL/kg LNI 2.8 plasma given 0, 3, and 6 dpi (N = 10)<br>3) 12 mL/kg LNI 2.8 plasma given 0, 3, and 6 dpi (N = 10)<br>4) No treatment (N = 10)	1)80%<br>2) 100%<br>3) 100%<br>4) 0%	[94]
		**HuMAb 25.6A**		

Hartley Guinea Pig	IP 1,000 pfu of GPA LASV Josiah	1) 30 mg/kg given after given 0, 3, and 6 dpi IP (N = 5)<br>2) Pooled untreated and human IgG1 controls (N = 18)	1) 100%<br>2) 6%	[101]
**HuMAb 2.9D**				
Hartley Guinea Pig	IP 1,000 pfu of GPA LASV Josiah	1) 30 mg/kg given after given 0, 3, and 6 dpi IP (N = 5)<br>2) Pooled untreated and human IgG1 controls (N = 18)	1) 100%<br>2) 6%	[101]
**HuMAb 8.9F**				
Hartley Guinea Pig	IP 1,000 PFU of GPA LASV Josiah	1) 30 mg/kg given after given 0, 3, and 6 dpi IP (N = 5)<br>2) Pooled untreated and human IgG1 controls (N = 18)	1) 100%<br>2) 6%	[101]
**HuMAb 12.1F**				
Hartley Guinea Pig	IP 1,000 PFU of GPA LASV Josiah	1) 30 mg/kg given after given 0, 3, and 6 dpi IP (N = 5)<br>2) Pooled untreated and human IgG1 controls (N = 18)	1) 100%<br>2) 6%	[101]
**HuMAb 37.7H**				
Hartley Guinea Pig	IP 1,000 PFU of GPA LASV Josiah	1) 30 mg/kg given after given 0, 3, and 6 dpi IP (N = 5)<br>2) Pooled untreated and human IgG1 controls (N = 18)	1) 100%<br>2) 6%	[101]
**HuMAb 37.2D**				
Hartley Guinea Pig	IP 1,000 PFU of GPA LASV Josiah	1) 30 mg/kg given after given 0, 3, and 6 dpi IP (N = 10)<br>2) Pooled untreated and human IgG1 controls (N = 18)	1) 90%<br>2) 6%	[101]
**HuMAb 19.7E**				
Hartley Guinea Pig	IP 1,000 PFU of GPA LASV Josiah	1) 30 mg/kg given after given 0, 3, and 6 dpi IP (N = 10)<br>2) Pooled untreated and human IgG1 controls (N = 18)	1) 90%<br>2) 6%	[101]
**HuMAb 37.2G**				
Hartley Guinea Pig	IP 1,000 PFU of GPA LASV Josiah	1) 30 mg/kg given after given 0, 3, and 6 dpi IP (N = 5)<br>2) Pooled untreated and human IgG1 controls (N = 18)	1) 80%<br>2) 6%	[101]
**HuMAb 10.4B**				
Hartley Guinea Pig	IP 1,000 PFU of GPA LASV Josiah	1) 30 mg/kg given after given 0, 3, and 6 dpi IP (N = 5)<br>2) Pooled untreated and human IgG1 controls (N = 18)	1) 40%<br>2) 6%	[101]
**HuMAb 25.10C**				
Hartley Guinea Pig	IP 1,000 PFU of GPA LASV Josiah	1) 30 mg/kg given after given 0, 3, and 6 dpi IP (N = 5)<br>2) Pooled untreated and human IgG1 controls (N = 18)	1) 30%<br>2) 6%	[101]
**HuMAb 36.1F**				
Hartley Guinea Pig	IP 1,000 PFU of GPA LASV Josiah	1) 30 mg/kg given after given 0, 3, and 6 dpi IP (N = 5)<br>2) Pooled untreated and human IgG1 controls (N = 18)	1) 20%<br>2) 6%	[101]

LNI = Log10 neutralization index = [Log10 (PFU in control)-Log10 (PFU in test serum)] (LNI determined based on challenge strain)(Jahrling 1983). IFA = Indirect fluorescent antibody. SQ = Subcutaneous. IP= Intraperitoneal. IV = Intravenous. DPI = Days post infection. N = group size. PFU = plaque forming units. GPA = Guinea pig adapted. LASV = Lassa virus.
